# Dispersion of hyperenhancement in late gadolinium enhancement cardiovascular magnetic resonance measured with Moran's I is associated with a decrement in LVEF 6 months after cardiotoxic chemotherapy

**DOI:** 10.1186/1532-429X-15-S1-P156

**Published:** 2013-01-30

**Authors:** Jennifer H Jordan, Craig A Hamilton, Ralph B D'Agostino, Julia Lawrence, Sujethra Vasu, W Gregory Hundley

**Affiliations:** 1 Internal Medicine (Cardiology), Wake Forest University School of Medicine, Winston Salem, NC, USA; 2 Biomedical Engineering, Wake Forest University School of Medicine, Winston-Salem, NC, USA; 3 Public Health Sciences, Wake Forest University School of Medicine, Winston Salem, NC, USA; 4 Internal Medicine (Hematology and Oncology), Wake Forest University School of Medicine, Winston Salem, NC, USA; 5 Radiology, Wake Forest University School of Medicine, Winston Salem, NC, USA

## Background

**Figure 1:**
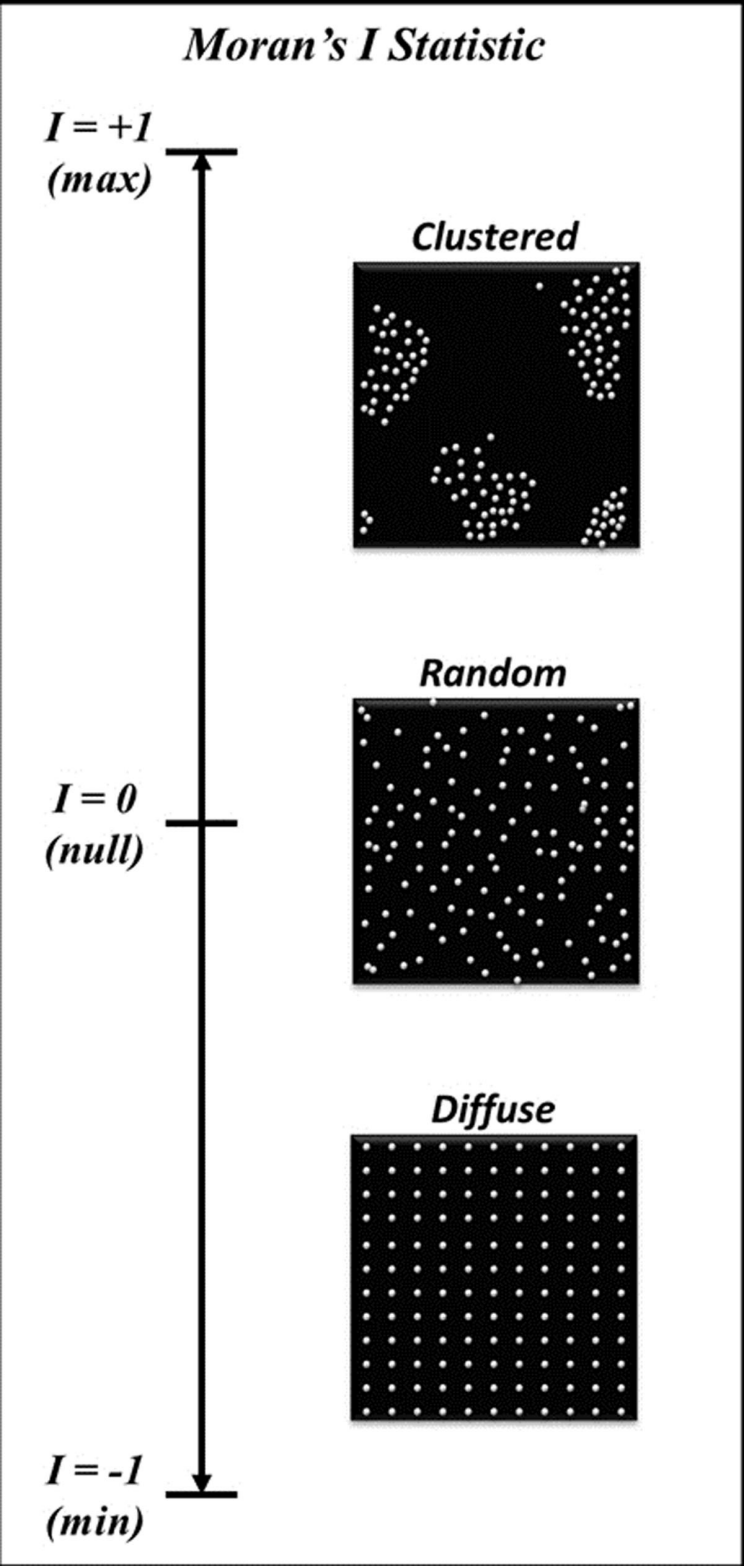
Illustration of the spatial autocorrelation statistic, Moran's I, which describes the distribution of values in a volume in relation to their difference from the mean value and proximity to other voxels of similar intensity value. Moran's I ranges from I=-1 (diffuse distribution) to I=+1 (clustered distribution) and I=0 represents random distribution in the volume of values different from the mean value. Each illustration in the image contains the same number of white dots (or mean value) but different patterns of distribution.

In animals and human subjects, an increase in background signal intensity observed on late gadolinium enhanced (LGE-SI) images is associated with a decrement in left ventricular ejection fraction (LVEF) during receipt of anthracycline chemotherapy. Moran's I statistic is a measurement of spatial dispersion of hyperenhanced voxels relative to the mean myocardial LGE-SI, ranging from highly clustered (I=+1) to highly diffuse (I=-1) (Figure 1). We hypothesize that a change in the distribution of hyperenhanced voxels (due to the development of high signal "micro clusters") is associated with a decrement in LVEF after cardiotoxic chemotherapy.

## Methods

We performed a prospective, extramurally-funded longitudinal cohort study of 51 participants (43 women, 8 men; aged 52±2 years) scheduled to receive 3 to 4 months of potentially cardiotoxic chemotherapy (anthracycline or trastuzumab) for treatment of breast cancer or hematologic malignancy. Before and then 3 and 6 months after chemotherapy initiation, participants underwent cardiovascular magnetic resonance (CMR) assessments of LVEF, LGE-SI, and Moran's I statistic determined by personnel blinded to participant identifiers and all other aspects of the analyses. Results were analyzed using paired Student's t-tests to test for a difference between baseline and subsequent examinations, and one-way ANOVA to test for trending change. All values are reported as mean ± standard deviation with p-values<0.05 considered statistically signficant.

## Results

**Figure 2:**
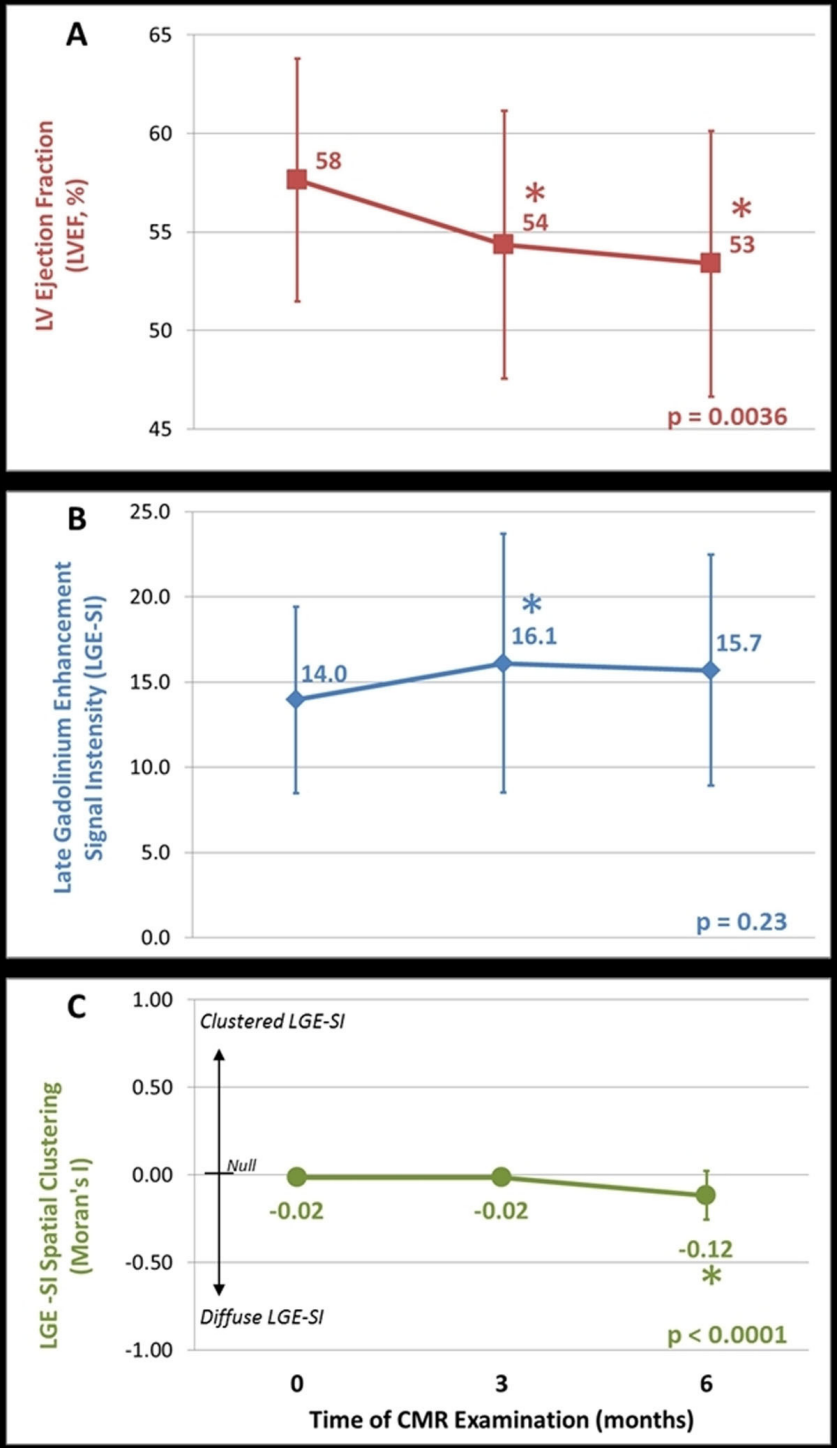
Longitudinal measurements of left ventricular ejection fraction (LVEF, Panel A), late gadolinium enhancement signal intensity (LGE-SI, Panel B), and LGE-SI pattern (Moran's I, Panel C) measured with 1.5T cardiovascular magnetic resonance prior to chemotherapy administration (month 0) and again 3 and 6 months after administration of cardiotoxic chemotherapy. Mean values ± standard deviation shown in each panel. Significant differences from baseline by paired t-tests with p-values<0.05 indicated by (*); p-value listed in each panel is for analysis of trend using one-way ANOVA.

37 participants were treated for breast cancer and 14 for hematologic malignancy. A declining LVEF from baseline (58±6%) was observed three months (54±7%) and six months (53±7%) after beginning chemotherapy (p<0.0001 for trend, Figure 2A). Mean LGE-SI, reflecting a change in myocardial T1 relaxation, increased from 14.0±5.5 at baseline to 16.1±7.6 three months after starting chemotherapy (p=0.03, Figure 2B) and remained elevated at 6 months (15.7±6.8, p=0.07 from baseline). At baseline and 3 months, the patterns of LGE-SI hyperenhancement (Moran's I statistic) showed random distribution (-0.02±0.02 and -0.02±0.01, respectively; p=0.91). Six months after chemotherapy initiation, myocardial LGE-SI hyperenhanced voxels became more diffusely distributed as shown in Figure 2C (I=-0.12±0.14, p<0.001).

## Conclusions

We observed that, six months after receipt of chemotherapy, increased late gadolinium enhancement signal intensity (LGE-SI) occurs in a diffusely distributed pattern within the myocardium concurrent with a declining LVEF. Moran's I statistic is a novel method to discriminate processes related to a diffuse increase in myocardial T1 (fibrosis, edema) from those related to a clustered increase in myocardial T1 (infarct); further investigations are warranted to study the utility of Moran's I statistic with T1 and T2 mapping.

## Funding

This work was supported in part by the National Institutes of Health grant R33CA12196 (Hundley), American Heart Association Predoctoral Fellowship 09PRE2210050 (Jordan), and a grant from the Susan G. Komen Foundation BCTR07007769 (Hundley).

